# Vital Force and the *Advance*

**Published:** 1884-07

**Authors:** P. P. Wells


### HOMŒOPATHIC PHYSICIAN,

A MONTHLY JOURNAL OF MEDICAL SCIENCE.

"If our school ever gives up the strict inductive method of Hahnemann, we are lost, and deserve only to be mentioned as a caricature in the history of medicine."—CONSTANTINE HERING.

Vol. IV. JULY, 1884. No. 7.

### VITAL FORCE AND THE "ADVANCE."

In No. 1, Vol. IV, of THE HOMŒOPATHIC PHYSICIAN we commented on a criticism of the *Medical Advance*, in which we found the existence of the vital force was universally "denied by modern scientists," and the editor of the *Advance* joined in this denial. In our comment we endeavored not only to show that a vital force *is*, but *what* it is and what it *does*. We were glad in believing we had made these points so plain that there need be no mistaking them; that we had shown them "*demonstrated*," in clear light, so that all might see them except the willfully blind. The *Advance* did not so see them and in the January number, 1884, gave us what was intended as a reply to our comment. But, as it failed to touch any one point of our argument or to set aside any one of our demonstrated facts, or, rather, facts self-demonstrated, we did not see that any reply to this was called for from us. In this failure, which was so complete, we recognized an abandonment by the *Advance* of argument in the case, while it confined itself to repeating for self and the "scientists," its denial of a vital force, and declaring the claim for the existence of such force was "unscientific." This it had done before, and it was not apparent how mere repetition of a false opinion gave it any additional strength or authority. This looked like giving up the argument and cleaving to negation.

In the *Advance* for May we find it still pursuing this course. In attempting an answer to a paper by our friend, Dr. B. Fincke, on this subject, it still clings to negation and adds testimony from Draper and Carpenter to sustain its position. Now, we knew before that these gentlemen and many like them had failed to recognize the *living facts* before their eyes and that they had denied that these facts prove that which they so clearly demonstrate—the existence of a force which produced them. We knew this. But what then? We cannot concede to these men, scientists though they be, power to annihilate facts or to set aside their natural relations of cause and effect by any negations of theirs, however earnest or often repeated. And we may add right here, when so-called scientists attempt this, verily "*the cobbler" has gone beyond " his last.*"

We return to this subject and the *Advance's* connection with it to correct, if we may, some of its apparent oversights in its attempted reply to Dr. Fincke, not because Dr. B. or Dr. F. are not abundantly capable of taking care of themselves, but because we have in some of its statements been placed in the excellent company of Dr. F. and Dr. B. and been held with them in fault for not having done some things which, we believe, have not only been done, but done in a manner and with conclusions not easily gainsaid. Says the *Advance*, p. 579, Vol. XIV :

> "What is exactly to be understood by the 'vital force' was not defined by Dr. Bayard, nor has it since been defined by his friends and defenders."

We thought we had made this sufficiently plain when we represented it as that "in the human organism which, while present in it, preserves its parts in integrity of tissue and function; which, when removed, these pass under the dominion of laws that reduce the whole to destructive dissolution." And further, we thought we had given a sufficiently explicit expression, and with sufficient "exactness," of what is to be understood by the "vital force" when we stated it to be the motive power of all functions.

It is not a new resort in criticism which charges with failure to do that which the writer has not only done, but perhaps done well, as was instanced in an Old School reviewer's attempt to criticise Dunham's *Homœopathy the Science of Therapeutics.* The reviewer charged the writer with having omitted to give in this work a statement of what the writer understood to be necessary to the constitution of a true science—the very thing the writer had done—not omitted—and had done so clearly.* But says the *Advance:*

---

*The principle on which this kind of criticism rests seems quite in harmony with the shrewd advice given by an elder lawyer to a younger, " When your client has no *defense,* abuse the plaintiff's counsel."

"If the existence of the vital force is so very 'self-evident,' it needs no argument to support it, and without much waste of words it might be demonstrated so that we could all see it. Now, will Dr. Bayard or Dr. Wells or Dr. Fincke please give us the much needed demonstration?"

This which the *Advance* so modestly yet earnestly seeks was fully presented to its attention in THE HOMŒOPATHIC PHYSICIAN in the paper of January 1st ult. as present in every function of every living organism, governing and executing those functions which in the absence of this force at once and wholly cease. Where function exists this force is present as its executor, and every performed function is a demonstration of its presence, deny this whoever may. It is just possible the *Advance* may have regarded itself as calling for a different kind of demonstration, "one that all can *see*." The above-quoted paragraph seems to more than hint that this is the kind it requires, and so, not having *seen* the thing itself, this is a sufficient reason for scientists and the *Advance* denying its existence. This is going one step further in the matter of proof of the existence of this force than did von Grauvogle when he refused to recognize it because he could not bring it into the experience of scientific experiment. He did not claim the right to *see* it, but only to deal with it as with elements of chemistry or physics in experiments appropriate to those sciences. This he would do or he would have none of it. Silly as this was, and silly because the reason could only have come from an entire failure to appreciate the difference in the nature of those sciences which render the experiments and demonstrations belonging to the one impossible to the other. And Dr. von Grauvogle would have these, or he would not accept the force. The *Advance* will go one step further and insist on *seeing* it—a little more silly only. Deny it, because you cannot see it? Will you be logical, and deny the existence of a *thought* for the same reason? No man has seen a thought but only the expression of it on the printed or written page or heard it in speech. So no man has seen, or can see, the vital force, but any man may see the clearest expression of it who will take the trouble to observe the operation of any of the functions of a living organism.* The one expression is not clearer than the other. If there be still any man who refuses to acknowledge the existence of this force because he cannot make it the subject of "scientific experiment," as did

*Has the *Advance seen* the *force* of gravitation? If not, will he therefore deny its existence? Has he *seen any* other natural force? If not, does he deny the existence of such force? If not, then why, for this reason, deny existence of a vital force?

von Grauvogle, then let him make a *thought* the subject of a like experiment, which if he fails to do, let him be at least a consistent skeptic, and deny that there is or ever has been any such thing as thought in the world. The one is no more ridiculous than the other. But further the *Advance:*

"If the vital force exists in living structures, surely our physiologists should take some cognizance of it."

No doubt this was their duty. But their neglect of this duty is not to be permitted to bring damage to truth and science, and especially to that science it was their duty to teach in its entirety, or to that truth which constitutes so important a factor in the philosophy of the divine law of healing. And it does not follow that their neglect of this duty annihilated the fact thus neglected. The omission goes far to prove their partial qualification as teachers of their specialty, or their inexcusable neglect of a most important, even a fundamental, principle of the science of which they were the professed teachers. Physiology we understand to be the science of life. These men who are found denying or neglecting the force here in question are teachers of this science. Did they teach the science of a life without force in it? If so, wherein was that they taught different from the death which is only an absence of this force, and therefore of life? Life without force is no life. This neglect or denial on their part is only an additional evidence, not now needed, how great blinding powers, prejudice, and pride may become, and into how great absurdities they may at times plunge men who in other matters may be leaders in general intelligence. P. P. WELLS.

